# Gaming disorder symptoms and psychiatric comorbidity from adolescence to early adulthood – a longitudinal study

**DOI:** 10.1007/s00787-026-03085-5

**Published:** 2026-06-13

**Authors:** Samuel Savander, Susanne Olofsdotter, Sofia Vadlin, Cecilia Åslund, Hans Arinell, Kent W. Nilsson, Johan Isaksson

**Affiliations:** 1 Department of Medical Sciences, Child and Adolescent Psychiatry Unit, Uppsala University, Uppsala, S-751 85 Sweden; 2 Centre for Clinical Research, Västmanland County Hospital Västerås, Uppsala University, Uppsala, Sweden; 3 CHAP, Department of Public Health and Caring sciences, Uppsala University, Uppsala, Sweden; 4 Division of Public Health, School of Health, Care and Social Welfare, Mälardalen University, Västerås, Sweden; 5 Centre of Neurodevelopmental Disorders (KIND), Centre for Psychiatry Research, Department of Women’s and Children’s Health, Karolinska Institutet & Stockholm Health Care Services, Region Stockholm, Sweden

**Keywords:** Comorbidity, Gaming disorder, Longitudinal, Trajectories, Psychiatric symptoms

## Abstract

Gaming is an increasingly common pastime activity. Gaming Disorder (GD) is characterized by excessive gaming habits, loss of control and priority of gaming resulting in negative consequences. Psychiatric symptoms have been suggested as an important factor affecting GD, yet longitudinal studies are limited. This study examined the temporal stability of GD symptoms and longitudinal comorbidity with psychiatric symptoms, from adolescence to adulthood. Self-ratings on symptoms of GD, attention-deficit/hyperactivity disorder (ADHD), depression, anxiety and conduct disorder (CD) were collected at four waves (W1: *n* = 1834, Mean age = 14.40; W2: *n* = 1642, Mean age = 17.37; W3: *n* = 1211, Mean age = 20.19; W4: *n* = 1066, Mean age = 22.93). Linear Growth Mixture Models were used to assess trajectories of GD symptoms. Generalized Linear Mixed Models and binary logistic regression were used for investigating associations between GD trajectories and psychiatric comorbidity. Three trajectories of GD symptoms were found, “Low Descending” (*n* = 1436), “Moderate Stable” (*n* = 211) and “Problematic Ascending” (*n* = 74). The “Moderate Stable” group rated more symptoms of ADHD, anxiety and depression, whereas the “Problematic Ascending” group rated more symptoms of ADHD, anxiety and CD, as compared to the “Low Descending” group. The “Moderate Stable” and the “Problematic Ascending” groups also had higher odds of reporting a diagnosed psychiatric condition. No sex-specificity were found between GD trajectories and psychiatric comorbidities, although males rated more GD symptoms. GD and psychiatric symptoms followed parallel developmental patterns, suggesting a reciprocal relationship. The findings stress the importance of identifying problematic gaming when assessing psychiatric symptoms, and vice versa, potentially important for targeted prevention and early intervention.

**Supplementary Information:**

The online version contains supplementary material available at https://doi.org/10.1007/s00787-026-03085-5.

## Introduction

Gaming has become a popular pastime activity and career-path, with gaming industry revenues steadily increasing and predicted to continue rising [1]. With an increase in popularity, and multiple avenues of excitement such as intermittent rewards and social features to incite more gaming, the risk of developing more hazardous habits may increase [2]. In response to an increasing population of individuals exhibiting more problematic gaming habits and a growing body of evidence suggesting clinical harm, Internet Gaming Disorder (IGD) was included as a condition recommended for further research within the Diagnostic and Statistical Manual of Mental Disorders 5th Edition (DSM-5), and Gaming Disorder (GD) was later recognized in the International Classification of Diseases 11th Revision (ICD-11) [3–6]. Henceforth, the term GD will be used unless specifically speaking about IGD, as GD is an established diagnosis in the ICD-11. Moreover, as gaming is not only limited to internet-based activities, the term “internet” in the DSM-5 terminology may be considered redundant, and we accordingly adopt the term “Gaming Disorder”. GD is characterized by an impaired control over gaming, prioritization of gaming over other activities, and continuation or escalation of gaming despite negative consequences, aspects also being covered in the definition of IGD by the DSM-5 [5, 6].

Evidence on the relationship between GD and other psychiatric conditions such as attention-deficit/hyperactivity disorder (ADHD), depression, anxiety and conduct disorder have been seen in cross-sectional studies in children, adolescents and adults [7–12]. However, only a limited number of studies have investigated the longitudinal relationships between GD and psychiatric comorbidity [9]. Psychiatric symptoms of depression, anxiety and symptoms of ADHD have been suggested in longitudinal data to be both risk factors for and a consequence of GD, as well as reciprocally associated with GD, while no longitudinal associations between GD and conduct disorder were reported [9, 13]. Most longitudinal studies have been limited to ≤ 2 years of follow-up (some up to five years), focusing on either children and adolescents, or adults, and rarely examine developmental changes in gaming habits [9]. Thus, the temporal order and direction of associations between GD and psychiatric symptoms remain unclear [10, 14].

Different eras in life could relate to different opportunities to engage in gaming and associated problematic gaming behaviors, and have a difference in psychiatric vulnerability factors [7–9]. Reported stability of GD ranges from 20 to 84%, depending on age and study duration, though no studies have followed participants for more than five years [9]. Sex is also an important factor to acknowledge in the study of GD and comorbid psychiatric symptoms, with males being more likely to exhibit problematic gaming, being diagnosed with conduct disorder and ADHD during childhood and adolescence, although these sex differences in ADHD diminish in adulthood [15–17]. In contrast, anxiety and depression is more common among females [17].

By utilizing longitudinal data spanning 10 years, this study investigated the following hypotheses that: (1) repeated ratings on GD symptoms during adolescence and early adulthood will result in subgroups of different trajectories with some stability across the waves, (2) subgroups with higher levels of GD symptoms will have higher levels of comorbid anxiety, depression, and ADHD, and that the relationship between GD symptoms and comorbidity will be reciprocal, and lastly, (3) there will be significant sex differences where males have a higher degree of GD symptoms and conduct problems whereas females have a higher degree of anxiety and depression symptoms, and the associations between different trajectories and comorbidity will differ by sex.

## Methods

### Participants and procedure

We used a cohort from the prospective study *the Survey of Adolescent Life in Västmanland*, *SALVe* [18]. In 2012, all adolescents (*N* = 4712) born in 1997 or 1999 and their parents were invited to participate through regular mail by completing a self-report questionnaire about the participants’ mental health and sociodemographic factors. The response rate for the first wave in 2012 was 40%, and since then three more waves have been completed (2015, 2018, 2021). Wave one had 1834 responders (mean age = 14.40, SD = 1.04; 55.6% females), wave two 1642 responders (mean age = 17.37, SD = 1.05; 58.5% females), wave three 1211 responders (mean age = 20.19, SD = 1.09; 62.2% females), and wave four 1066 responders (mean age = 22.93, SD = 1.05; 64.4% females).

## Measures

Symptoms of GD was assessed using The Gaming Addiction Identification Test (GAIT), a 15-item scale covering playtime (2 items) and seven DSM-5 IGD symptoms (13 items) while also encompassing the criteria for GD set forth by the WHO, with items regarding increased prioritization (e.g., “I play more often and/or for longer periods now compared with previously”), lack of control (e.g., “It often happens that I play more than I had planned”), and functional impairment (e.g., “My health has been negatively affected by my playing”) [5, 6, 18]. The 15 items are rated on a five-point scale from 0 (disagree) to 4 (completely agree). The scale has been validated and shows a high internal consistency and a high concurrent validity with a similar established questionnaire [18]. Higher scores indicate more problematic gaming and a score of ≥ 19 has been used as a cut-off [19]. After the first wave, this scale was revised to include 17 items encompassing all nine symptom criteria for IGD, however, this was not utilized in this study as to not divert from wave 1 [18].

ADHD symptoms were measured using a shorter version of the World Health Organization Adult ADHD Self-Report Scale screener (ASRS-S) and its adolescent version ASRS-A-S [20, 21]. Both consist of 6 items covering core ADHD domains, with ASRS-A-S having simpler wording. Items are scored on a scale ranging from 0 (never) to 4 (very often), where higher scores indicate more ADHD symptoms. The ASRS-S has showed a clinical diagnosis concordance of 0.87, as well as a good internal consistency in a Swedish sample of adult psychiatric patients, and in a Swedish sample of adolescents using the corresponding short version for adolescents ASRS-A-S [20–22].

Anxiety symptoms were measured using Spence´s Children Anxiety Scale (SCAS) for wave 1–2 when the participants were still under 18 years of age, and the Adult Anxiety Inventory (AAS-15) in wave 3–4 to accommodate their age [23, 24]. SCAS includes 44 items covering generalized anxiety, fears of physical injury, social anxiety, separation anxiety, panic disorder and agoraphobia, on a four-point Likert scale ranging from 0 (never) to 3 (always) where a higher score indicates higher level of anxiety symptoms. 38 items are counted towards the total score whereas six items are control questions. The scale has shown an excellent internal consistency in a Swedish sample of adolescents [23]. The AAS-15 includes 15 items rated on the same scale as SCAS, assessing generalized anxiety, social anxiety and panic disorder, and has been shown to have an excellent internal consistency in Swedish sample [24]. Due to having two different scales, the scores have been converted to percentage in order to establish a valid temporal relationship.

Depressive symptoms were measured using the Depression Self-Rating Scale (DSRS) for wave 3–4 and its adolescent version (DSRS-A) for wave 1–2, to accommodate for age. DSRS is used to screen for major depressive disorder based on the DSM, and the original validation study on DSRS indicated a sensitivity of 86% and a specificity of 75% compared with an expert rated diagnosis [25]. DSRS-A has been validated in a community-based sample of adolescents and in child- and adolescent psychiatric outpatients in Sweden, reporting a moderate concurrent validity and moderate diagnostic accuracy [26]. Participants reported depressive symptoms over the past two weeks on 15 yes/no items. We created a depression index by summating the reported symptoms. Although some items on the DSRS refer to the same symptom, we counted each symptom only once, which produced a depression index range of 0–9, with a higher score indicating more depressive symptoms [27].

Conduct Disorder symptoms were measured at waves 1–2 with a 26-item questionnaire based on a questionnaire by Andershed, Kerr and Stattin [28], supplemented with common disruptive behaviors [29]. The questionnaire has shown good internal consistency [27]. For this study, the answers were dichotomized (yes/no) to correspond with wave 3 and 4, which utilized a shorter version using 15 items regarding delinquency and violence which was scored binary, also based on Andershed, Kerr and Stattin [28] and has shown acceptable internal consistency [30]. Due to having two different scales during wave 1–2 and wave 3–4, the values were converted to percentages trying to create a valid temporal relationship.

Psychiatric diagnoses were measured using self-reported data on dichotomous variables at wave 3–4 where participants answer (Yes/No) on questions regarding if they have received a specific diagnosis from a licensed health professional.

We used the occupational status of the participants’ parents at wave 1 as a proxy for socioeconomic status (SES). SES was categorized as 1 = both parents were working or studying, and 0 = other (i.e., either or both parents were not working or studying).

### Statistical analysis

Analyses were performed using SPSS v.29 with p values < 0.05 considered to be significant. Generalized Linear Models were used to compare GD symptoms and co-occurring psychiatric symptoms between the four waves, whereas independent samples t-test were used for analyzing sex differences at each wave in relation to GD symptoms and co-occurring psychiatric symptoms.

To test hypothesis 1, we employed a latent growth mixture model (LGMM) using MPlus software by Muthen & Muthen [31] to identify longitudinal trajectories of GD symptoms. The LGMM utilizes a robust full information maximum-likelihood (FIML) estimation procedure, it provides unbiased estimates using all available observations, in this case we chose to limit our estimates to participants with at least two measures of GD symptoms. The total number of participants available for the MPlus analyses was *n* = 1721. LGMM-analyses with two to six classes were estimated and compared based on the interpretability of the model, the number of participants in the classes, and a set of fit indices by Muthen [32]; Akaike’s information criterion (AIC), the Bayesian information criterion (BIC), the sample size adjusted BIC (SSA-BIC), the Lo–Mendell–Rubin likelihood ratio test (LMR LRT).

For the second and third hypotheses, we used Generalized Linear Mixed Modeling (GLMM) with a negative binomial distribution since our data was over-dispersed and showed greater variance than means, using the residual method for approximating the degrees of freedom, also adjusting for sex. AIC was used to evaluate fit. Interaction was tested for trajectories by wave, trajectories by sex, and a three-way interaction between trajectories, wave and sex. A One-Way ANOVA was used to investigate whether SES differed between the different trajectories.

Binary logistic regression was used to assess whether the different trajectories increased the odds of an acquired diagnosis during their life of ADHD, anxiety or depression at wave 3–4, while also adjusting for sex and age. The diagnoses were self-reported (yes/no). No questions regarding diagnosed GD or conduct disorder were present in the battery. Goodness of fit was tested using the Hosmer and Lemeshow test, and the Omnibus Test of Model Coefficients was used to evaluate model significance.

## Results

As shown in Supplementary Fig. 1, the number of data points varied across waves and questionnaires, since not all participants answered the full battery. Descriptive analyses were based on the pre-LGMM sample (*N* = 1905). The analyzes run with trajectories utilizes the datapoints post data manipulation for LGMM (*N* = 6884). Attrition analysis comparing those with at least two measures of GD symptoms available for the MPlus analyses with those having only one rating of GD symptoms showed no significant difference in rated psychiatric symptoms, however, those lacking data from at least two waves rated higher GD symptoms (t = 2.27, *p*=.03, *d*=0.25), were predominantly males (*χ2* = 16.84, *p*<.001, *ϕ* = 0.10), and had lower SES (*χ2* = 8.25, *p*=.006, *ϕ*=-0.07).

## GD symptoms and associated psychiatric conditions

Table 1 presents the mean values across all study variables by wave and sex. Significant differences emerged across waves: GD ratings decreased with age, while anxiety and depressive symptoms increased with age. Symptoms of ADHD and conduct disorder varied between waves with the highest ratings at wave 2. Males had higher ratings on GD, and conduct problems, while females consistently recorded higher on depressive symptoms and anxiety.

**Table 1:** Means (M) and standard deviation (SD), as well as difference between males and females with corresponding effect sizes, and difference between waves, for the study variables

		Gaming disorder symptoms	ADHD symptoms	Anxiety symptoms%	Depressive symptoms	Conduct disorder symptoms%
Wave 1						
M(SD)	All	6.58 (8.30)	6.99 (4.37)	.16 (.11)	1.88 (2.21)	.10 (.10)
	Female	3.49 (5.90)	7.14 (4.37)	.20 (.12)	2.23 (2.39)	.09 (.09)
	Male	10.40 (9.20)	6.80 (4.36)	.12 (.09)	1.44 (1.88)	.12 (.11)
Sex differences		t=18.48***	t=1.63	t=15.04***	t=7.98***	t=6.94***
		*d* =.92	*d* =.08	*d* =.69	*d*=.37	*d* =.34
Wave 2						
M(SD)	All	5.52 (7.23)	9.34 (4.78)	.20 (.13)	2.90 (2.60)	.13 (.12)
	Female	2.81( 4.97)	9.92 (4.84)	.25 (.14)	3.59 (2.64)	.11 (.11)
	Male	9.32 (8.13)	8.51 (4.57)	.13 (.01)	1.93 (2.22)	.15 (.14)
Sex differences		t=18.50*** *d* =1.01	t=5.92*** *d* =.30	t=19.83*** *d* =.94	t=13.80*** *d* =.67	t=6.33*** *d* =.33
Wave 3						
M(SD)	All	3.57 (6.08)	7.84 (4.79)	.29 (.21)	3.10 (2.84)	.04 (.09)
	Female	1.97 (4.21)	8.25 (4.84)	.33 (.21)	3.64 (2.85)	.03 (.07)
	Male	6.18 (7.59)	7.17 (4.63)	.21 (.19)	2.21 (2.61)	.06 (.11)
Sex differences		t=10.87*** *d* =.73	t=3.85*** *d* =.23	t=10.76*** *d* =.62	t=8.93*** *d* =.52	t=5.61*** *d* =.37
Wave 4						
M(SD)	All	3.21 (5.94)	8.77 (5.23)	.32 (.22)	3.37 (2.87)	.03 (.07)
	Female	1.78 (4.15)	9.18 (5.29)	.36 (.22)	3.80 (2.90)	.02 (.06)
	Male	5.82 (7.59)	8.02 (5.06)	.24 (.20)	2.59 (2.63)	.03 (.08)
Sex differences		t=9.56*** *d* =.72	t=3.52*** *d* =.22	t=8.87*** *d* =.57	t=6.94*** *d* =.43	t=2.26* *d* =.15
All waves						
Wave difference *χ2*		211.46 ***	234.03***	788.53***	292.94***	890.35***
		1>2,3,4	2>4,3,1	4>3,2,1	4>3,2,1	2>1,3,4
		2>3,4	4>3,1	3>2,1	3>2,1	1>3,4
			3>1	2>1	2>1	3>4

*=*p* <.05 ***=*p* <.001

### Trajectories of GD symptoms

**Fig. 1:**
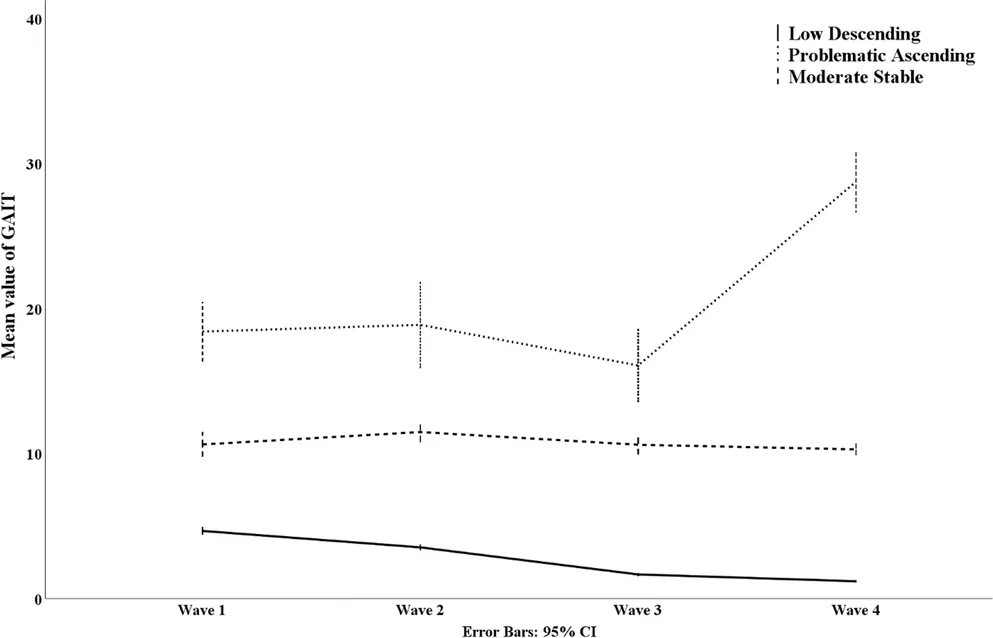
Trajectories of gaming disorder symptoms measured with GAIT from adolescence to early adulthood

Five different models were run for trajectories (see Supplementary Table 1 for fit indices), where the solution for three classes were chosen since it showed the optimal fit for the analyses. The solution with five classes demonstrated somewhat better fit, however, the classes became too small, why the three-class solution was chosen. Hence, three distinct trajectories of long-term GD symptoms from wave 1–4 were identified (Fig. 1). The majority (83.4%; *n* = 1436, 64.2% females) was characterized by low ratings on GD symptoms descending across all waves (“Low Descending”, intercept 6.33, linear slope − 1.37 (0.066), *p*<.001). This group was followed by a group (12.3%; *n* = 211, 28.9% female) characterized by stable intermediate GD symptom ratings in comparison to the other two groups in the study (“Moderate Stable”, intercept 12.76, linear slope − 0.46 (0.25), *p*=.07), and a group (4.3%; *n* = 74, 13.5% female) characterized by persistently high GD symptoms values bordering on problematic use with a marked increase at wave 4 (“Problematic Ascending”, intercept 15.07, linear slope 3.06 (0.70), *p*<.001). SES did not differ between groups and was therefore excluded from subsequent analyses.

## Trajectories of GD symptoms and co-occurrent psychiatric symptoms

We ran four models for all symptom groups: (1) trajectories, waves, and their interaction; (2) adding sex; (3) adding the trajectory×sex interaction; and (4) adding the trajectory×wave×sex interaction. The third and fourth models did not improve model fit. Supplementary Table 2 presents AIC, model significance, and random effects.

**Fig. 2:**
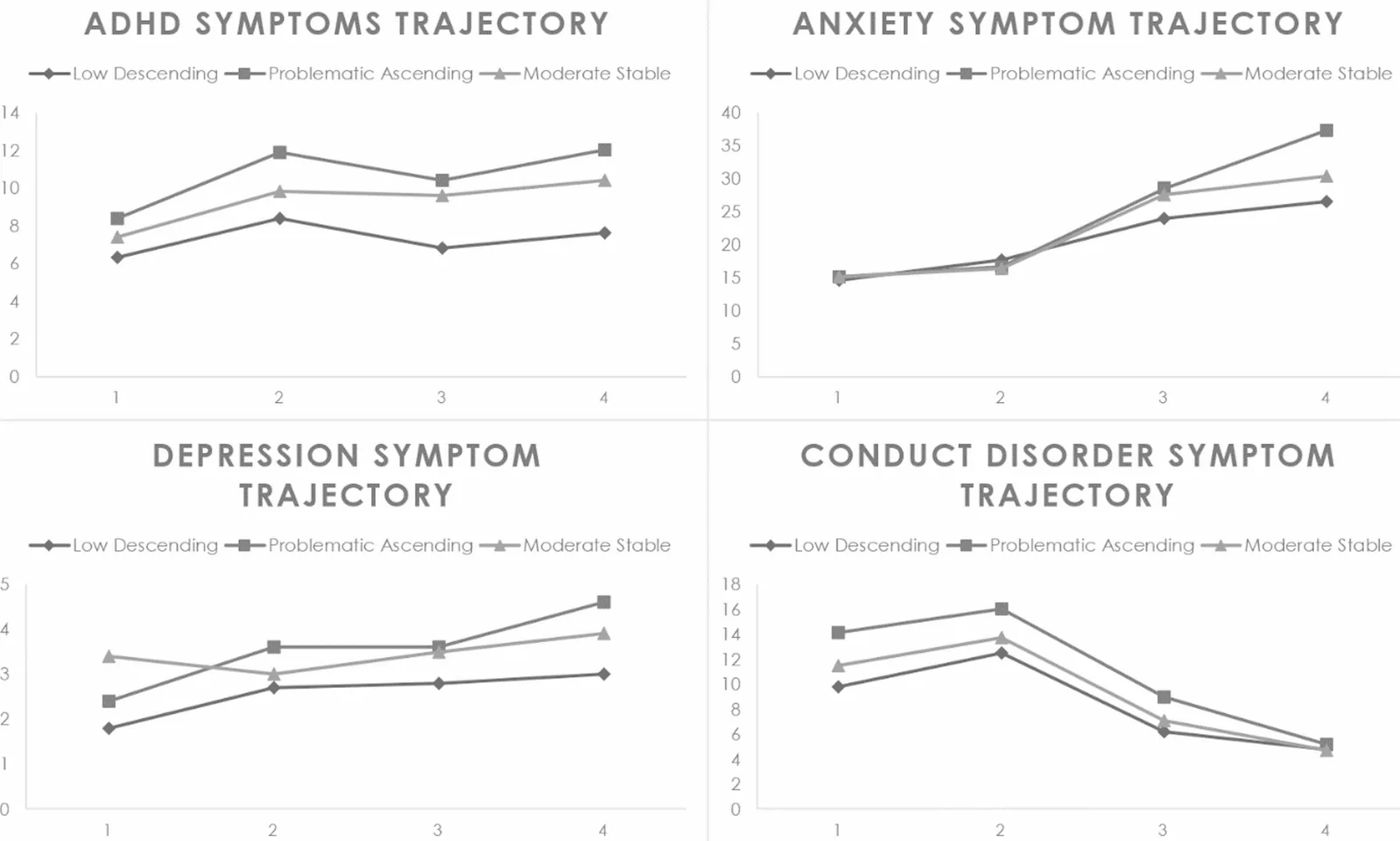
Trajectories of gaming disorder symptoms within each of the psychiatric symptom ratings

Table 2 shows the results of the second model which showed the best overall estimation of prediction error. The “Moderate Stable” group and the “Problematic Ascending” group had significantly higher symptom ratings for ADHD and anxiety than the “Low Descending” group. The “Moderate Stable” group also had significantly higher symptom ratings for depression, whereas the “Problematic Ascending” group rated significantly higher for conduct disorder. Wave 2, 3 and 4 had significantly higher symptom ratings for every condition, except for conduct disorder in wave 3 and 4, with lower ratings. Female ratings were significantly higher for ADHD, anxiety and depression symptoms, whereas for males on conduct disorder symptoms. For ADHD symptoms, a wave by trajectory interaction could be found within the “Moderate Stable” group for wave 3 and 4, indicating that the age of rating and a moderate amount of gaming interacts positively with higher ratings of ADHD symptoms compared to the “Low Descending” group. For anxiety, there was a significant interaction between the “Problematic Ascending” group and wave 4, indicating that the age of rating and problematic gaming interacts positively with higher ratings of anxiety symptoms compared to the “Low Descending” group (Table 2; Fig. 2).

**Table 2:** Symptoms of ADHD, anxiety, depression and conduct disorder regressed on trajectories of gaming disorder symptoms and sex

	ADHD (*n* = 5519)	Anxiety (*n* = 5529)	Depression (*n* = 5554)	Conduct Disorder (*n* = 5548)
	b(SE)	b(SE)	b(SE)	b(SE)
Trajectories *(Ref = Low Descending)*				
Moderate Stable	0.15 (0.05)***	0.27 (0.06)***	0.26 (0.08)**	0.16 (0.12)
Problematic Ascending	0.28 (0.08)***	0.37 (0.10)***	0.26 (0.13)	0.36 (0.18)*
Waves *(Ref = 1)*				
2	0.29 (0.02)***	0.18 (0.03)***	0.39 (0.04)***	0.24 (0.06)***
3	0.07 (0.03)**	0.47 (0.03)***	0.45 (0.04)***	− 0.47 (0.06)***
4	0.19 (0.03)***	0.57 (0.03)***	0.50 (0.05)***	− 0.71 (0.07)***
Wave×Trajectory				
2×Moderate Stable	0.00 (0.07)	− 0.12 (0.08)	− 0.15 (0.12)	− 0.07 (0.16)
2×Problematic Ascending	0.06 (0.11)	− 0.08 (0.13)	0.05 (0.19)	− 0.12 (0.27)
3×Moderate Stable	0.20 (0.07)**	0.10 (0.08)	− 0.04 (0.12)	− 0.03 (0.17)
3×Problematic Ascending	0.14 (0.13)	0.15 (0.16)	− 0.03 (0.22)	0.01 (0.32)
4×Moderate Stable	0.16 (0.07)**	0.27 (0.06)	0.01 (0.12)	− 0.18 (0.18)
4×Problematic Ascending	0.17 (0.14)	0.37 (0.10)*	0.17 (0.24)	− 0.28 (0.36)
Sex *(Ref=Male)*				
Female	0.18(0.02)***	0.64(0.03)***	0.48(0.03)***	− 0.28 (0.04)***

Based on Model two with wave, trajectory and sex, as well as an interaction between wave and trajectory. *=*p*<.05 **=*p*<.01 ***=*p*<.001

## Trajectories of GD symptoms and odds of a self-reported diagnosis in wave 3 and 4

As shown in Table 3, the “Problematic Ascending” group had higher odds of self-reporting “any diagnosis” and ADHD in early adulthood, while those belonging to the “Moderate Stable” group had a higher odds ratio of self-reporting ADHD, anxiety and depression, as well as “any diagnosis”. Females were more likely to self-report anxiety, depression or “any diagnosis”.

**Table 3:** Self-reported diagnoses at wave 3 or 4 (*n *= 1383) regressed on trajectories of gaming disorder symptoms, sex and age

	Diagnosis OR (95% CI)			
	ADHD	Anxiety	Depression	Any diagnosis
	(*n* = 79)	(*n* = 158)	(*n *= 152)	(*n *= 296)
Gaming *(Ref=Low Descending)*				
Moderate Stable	2.21	2.24	2.51	2.53
	(1.21,4.01)**	(1.39,3.61)***	(1.59,3.97)***	(1.75,3.65)***
Problematic Ascending	3.07	1.60	1.42	2.93
	(1.12,8.43)*	(.54,4.75)	(.48,4.16)	(1.48,5.78)**
Sex*(Ref=Male)*				
Female	1.50	3.55	2.38	2.09
	(.90,2.52)	(2.28,5.51)***	(1.58,3.58)***	(1.54,2.84)***
Age	1.02	.99	1.00	1.02
	(.82,1.27)	(.84,1.16)	(.85,1.17)	(.90,1.15)
Nagelkerke R^2^	.02	.06	.04	.04
Hosmer and Lemeshow*χ2*	df(8)=7.15	df(8)=3.79	df(8)=4.45	df(8)=6.47
Omnibus Test of Model*χ2*	df(4)=40.26***	df(4)=40.26***	df(4)=25.90***	df(4)=39.90***

Hosmer and Lemeshow Test shows adequate fit since *p*>.05

Omnibus Test of Model shows good fit since *p*<.05

*=*p*<.05, **=*p*<.01, ***=*p*<.001

## Discussion

In this longitudinal study, following a general population from adolescence to adulthood on GD symptoms and co-occurrent psychiatric symptoms, we identified three distinct trajectories of GD symptoms. The Moderate Stable group showed stable GD symptoms across time, the Problematic Ascending group showed stable high levels with a sharp increase in early adulthood, and the Low Descending group showed low symptoms early on which were further attenuated over time. Psychiatric symptoms followed these trajectories, with the “Moderate Stable” group and the “Problematic Ascending” group having higher ratings compared to the “Low Descending” group, indicating a reciprocal relationship. The “Moderate Stable” and the “Problematic Ascending” groups also had higher odds of having reported psychiatric diagnoses in adulthood. Males rated higher GD symptoms and conduct disorder symptoms, while females reported more ADHD, anxiety and depression symptoms; however, no interaction effects were found regarding sex and trajectories on psychiatric symptoms.

The model with three distinct trajectories of long-term GD symptoms from wave 1–4 was identified as having the best fit. There was a temporal stability primarily across the “Moderate Stable” group, with mean values approximate to one standard deviation above the sample average of the whole population. Previous research has reported dimensional stability for GD, where lower ratings yield lower ratings at follow-up, higher ratings also adhere to this relationship [33]. The “Low Descending” group’s decreasing symptoms may reflect protective factors in adolescence, whereas the “Problematic Ascending” group showed an escalation from levels bordering on problematic gaming according to the proposed cut-off [19], to exceeding it in the final wave, suggesting that high GD symptoms in youth may develop into problematic or addictive use in early adulthood. There was also a sex difference between the trajectories. The “Low Descending” group consisted predominantly of females (approximately a 2:1 female-to-male ratio), whereas the “Moderate Stable” and “Problematic Ascending” groups consisted predominantly of males (approximately 1:3 and 1:6, respectively). This pattern aligns with prior research showing higher GD prevalence in males [15, 34]. Although, our data is more skewed than previous research, with Stevens et al. [34, 35] approximating a 1:2.3 females-to-males’ ratio for GD. Even though there are different ratios of sex in our trajectories, the absence of significant sex interactions indicates that the developmental relationship between GD and psychiatric symptoms is similar for both sexes.

GD symptoms and psychiatric symptoms followed each other across the waves, supporting our hypothesis and prior research [9]. The “Low Descending” group had the lowest ratings for all psychiatric symptoms, while the “Moderate Stable” group and the “Problematic Ascending” group had higher symptom ratings for ADHD and anxiety, the “Moderate Stable” for depression, and the “Problematic Ascending” group for conduct disorder. Further, the “Moderate Stable” group had higher odds of reporting a diagnosis of ADHD, depression and anxiety, while the “Problematic Ascending” group had higher odds of reporting ADHD, compared to the “Low Descending” group, although with small variance explained. We have no data on motivations for gaming in this study, but psychiatric conditions such as anxiety and depression are correlated to escapism from negative sensations or situations, which have also been linked to developing GD [36, 37].

Regarding ADHD, research have indicated that impulsivity and inattention increase the risk of problematic addictions, as well as bidirectional relationships where ADHD increases gaming activities and gaming activities exacerbate ADHD symptoms [38]. Overall, our results suggest reciprocity rather than unidirectionality, where GD and psychiatric symptoms appear to reinforce one another over development, consistent with previous studies [9]. However, we did find interaction effects regarding waves and trajectories within symptoms of ADHD and anxiety, where the “Moderate Stable” GD group had a steeper increase in ADHD symptoms from adolescence to adulthood, although the “Problematic Ascending” group had the highest ADHD ratings, and where the “Problematic Ascending” group had a more pronounced increase in anxiety from adolescence to young adulthood, indicating that more GD symptoms in adolescence and early adulthood may trigger anxiety in adulthood.

Consistent with earlier research [15–17], females reported higher symptoms (and diagnoses) of anxiety and depression, while males scored higher on GD and conduct disorder, across all waves. These findings align with sex-related patterns, where females are more likely to internalize their emotions, whereas males tend to externalize, leading to different behaviors and conditions [17]. Interestingly, for ADHD, no sex difference regarding reported clinical diagnosis were found, although females rated higher levels of symptoms from late adolescence to adulthood. This is somewhat unexpected as ADHD is more common among boys, however, our finding may reflect diagnostic biases where ADHD is more often recognized in boys during childhood, whereas in females diagnoses occur later and with higher comorbidity and health care utilization [39].

### Strengths and limitations

Our study has several strengths, including the use of a large sample followed across 10 years, which is rarely done, and with an even sex distribution. However, several limitations should be mentioned. All measures were self-reported, raising risks of recall and reporting bias. Clinical interviews or data from medical journals would strengthen validity. We aimed to include symptoms of other addictions in our pre-registration of the study, including alcohol consumption, drug use and gambling, however, we realized that this scope would be too broad and chose to exclude those variables.

We switched the questionnaires for anxiety, depressive symptoms and conduct disorder when the participants reached adulthood to accommodate for development, which may negatively affect the comparative analysis. The version of GAIT used in this study only captured seven of the nine proposed criteria for IGD, not measuring deception or escapism. The reason for this was that the revised version of GAIT was not developed when the study started, and we prioritized to have the same version across all waves in order to more properly gauge the temporal relationship. Further, the GAIT was originally developed to assess symptoms consistent with IGD, as conceptualized prior to the introduction of GD in the ICD-11. However, the core symptom domains assessed by GAIT overlap with the diagnostic criteria for GD, and in the emerging literature, GD is increasingly regarded as a broader and more appropriate diagnostic construct than IGD [40].

Attrition across waves may introduce selection bias since those that drop-out often differ on several characteristics from those retained, but loss to follow-up rarely affects estimates of any found association [41]. In the present study, we choose to do a complete-case analyses, since methods for adjusting loss of cases, such as multiple imputation, involve assumptions that the data is missing at random. Given the size of our population, we believe that imputation is not necessary, and when computing the trajectories, we could include those with at least two data-points of GD data for inclusion. The attrition analysis showed no difference in psychiatric symptoms, however the mean value for GD symptoms were higher in the discarded population which could have influenced size of the “Problematic Ascending” group.

Our data was skewed where the majority of our participants in all samples scored low ratings on the variables, which was expected since we are measuring symptoms of psychiatric conditions within a non-clinical population. Together with the sex dispersion within the “Problematic Ascending” group, which was highly skewed towards males (86.4%), it is hard to draw any firm conclusion regarding sex-specificity in the association with other psychiatric conditions for this group. Including social media, which has been suggested to be included in future diagnostic manuals [42], might affect this relationship as studies indicate that females peruse social media more than males. We used parental occupation as a proxy for SES, parental education or familial income could have added additional information since these has been associated with GD [43].

## Conclusions

Gaming is a common pastime activity and even career-path today, but gaming is not without risk, why there is increasing focus within the scientific community towards both positive and negative aspects of gaming. In the present longitudinal study, GD symptoms and psychiatric symptoms followed each other across development for both males and females, indicating a reciprocal relationship. The findings stress the importance of identifying problematic gaming when assessing psychiatric symptoms, and vice versa. The findings are potentially important for targeted prevention and early intervention. Given that gaming can have both negative and positive effects it is also important to look at the motives and reasoning for gaming which we have not done in this study. For the future, it would be interesting to do similar studies in high-risk populations and important to include measures of social media usage.

## Supplementary Information

Below is the link to the electronic supplementary material.

Supplementary Material 1

Supplementary Material 2

Supplementary Material 3

## Data Availability

The dataset is not available because of regulations in the ethical approval. Request to access should be directed to kent.nilsson@regionvastmanland.se. Relevant data is included in the manuscript.
