# Supplementary material for: Gaming disorder symptoms and psychiatric comorbidity from adolescence to early adulthood – a longitudinal study
Source: Eur Child Adolesc Psychiatry. 2026 Jun 13;35(10):3179–88. doi: 10.1007/s00787-026-03085-5 (PMC13696595; doi:10.1007/s00787-026-03085-5)
Supplement: Supplementary file 1 [file 787_2026_3085_MOESM1_ESM.pdf]

Supplementary Table 1 Fit indices for two to six-class latent growth mixture models of gaming disorder symptoms.

Number of classes	AIC	BIC	SSA-BIC	Entropy	LMR LRT test value	Bootstrapped LRT *p*-value
Two	35062.33	35127.74	35089.62	.934	694.03	<.001
Three	34690.95	34772.71	34725.06	.875	361.22	<.001
Four	34696.95	34795.07	34737.88	.898	0	1.00
Five	34380.82	34395.28	34328.56	.863	174.33	<.001
Six	34299.89	34430.71	34354.46	.867	0	1.00

Note: Akaike's information criterion (AIC), the Bayesian information criterion (BIC), the sample size adjusted BIC (SSA-BIC), the Lo–Mendell–Rubin likelihood ratio test (LMR LRT).
