# Supplementary material for: Gaming disorder symptoms and psychiatric comorbidity from adolescence to early adulthood – a longitudinal study
Source: Eur Child Adolesc Psychiatry. 2026 Jun 13;35(10):3179–88. doi: 10.1007/s00787-026-03085-5 (PMC13696595; doi:10.1007/s00787-026-03085-5)
Supplement: Supplementary file 2 [file 787_2026_3085_MOESM2_ESM.pdf]

Supplementary Table 2 Summary of fit in models 1 through 4.

		ADHD symptoms	Anxiety symptoms	Depression symptoms	Conduct Disorder symptoms
			Model 1		
AIC		9816.51	11887.36	15332.53	18329.29
Model Significance		F(11, 5507) = 22.44***	F(11, 5517) = 49.11***	F(11, 5542) = 18.82***	F(11, 5536) = 31.03***
Random effects					
	b(SE)	.097(.008)	.19(.013)	.00	1.51E-8
	Z	12.76***	14.53***	-	-
			Model 2		
AIC		**9753.46**	**11353.19**	*15158.09*	18197.28
Model Significance		**F(12, 5506) = 25.44*** **	**F(12, 5516) = 92.73*** **	*F(12, 5541) = 37.52****	F(12, 5535) = 32.68***
Random effects					
	b(SE)	**.093(.007)**	**.14(.010)**	*1.18E-11*	1.34E-7
	Z	**12.55*** **	**13.18*** **	-	-
			Model 3		
AIC		*9755.97*	*11356.70*	**15155.61**	*18184.86*
Model Significance		*F(14, 5504) = 21.99****	*F(14, 5514) = 79.60****	**F(14, 5539) = 32.02*** **	*F(14, 5533) = 28.22****
Random effects					
	b(SE)	*.093(.007)*	*.14(.011)*	**1.32E-11**	*1.34E-7*
	Z	*12.55****	*13.20****	**-**	-
			Model 4		
AIC		9771.20	11360.49	15160.21	**18128.13**
Model Significance		F(23, 5495) = 13.97***	F(23, 5505) = 48.84***	F(23, 5530) = 20.00***	**F(23, 5524) = 16.91*** **
Random effects					
	b(SE)	.093(.007)	.139(.010)	2.38E-11	**1.34E-7**
	Z	12.60***	13.20***	-	**-**

Note: **Bold** indicates best model fit. *Cursive* indicates second best model fit. *** = p<.001. Model 1 = Wave, Trajectories, Wave*Trajectory; Model 2 = Wave, Trajectories, Wave*Trajectory, Sex; Model 3 = Wave, Trajectories, Wave*Trajectory, Sex, Trajectory*Sex; Model 4 = Wave, Trajectories, Wave*Trajectory, Sex, Trajectory*Sex, Wave*Trajectory*Sex.
