# Supplementary material for: Gaming disorder symptoms and psychiatric comorbidity from adolescence to early adulthood – a longitudinal study
Source: Eur Child Adolesc Psychiatry. 2026 Jun 13;35(10):3179–88. doi: 10.1007/s00787-026-03085-5 (PMC13696595; doi:10.1007/s00787-026-03085-5)
Supplement: Supplementary file 3 [file 787_2026_3085_MOESM3_ESM.pdf]

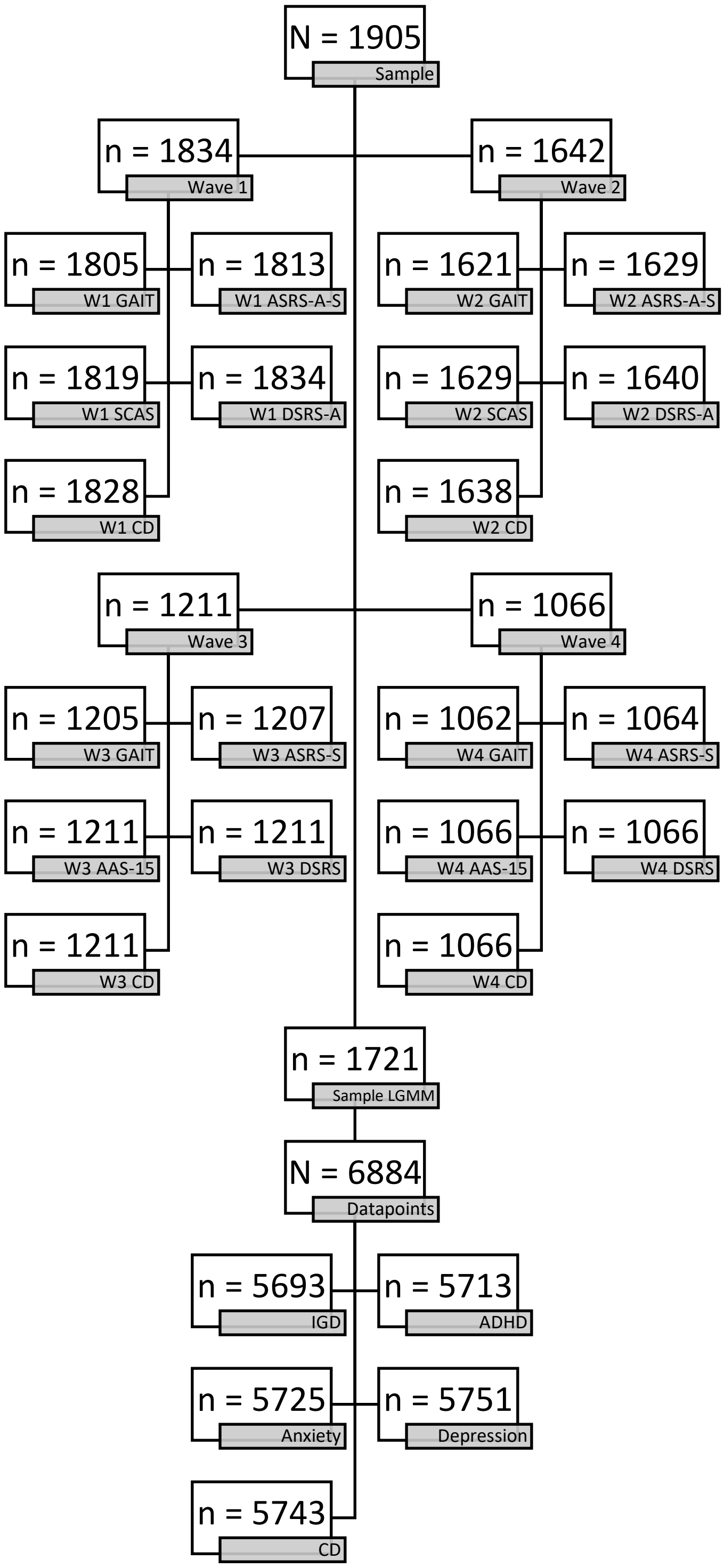

Supplementary Figure 1 Depiction of the number of participants and datapoints available for all different variables across all waves.
